# Follow-Up Survey of the Impact of COVID-19 on People Living with HIV during the Second Semester of the Pandemic

**DOI:** 10.3390/ijerph18094635

**Published:** 2021-04-27

**Authors:** Joseph Nelson Siewe Fodjo, Edlaine Faria de Moura Villela, Stijn Van Hees, Pieter Vanholder, Patrick Reyntiens, Robert Colebunders

**Affiliations:** 1 Global Health Institute, University of Antwerp, 2610 Wilrijk, Belgium; stijn.vanhees@uantwerpen.be (S.V.H.); robert.colebunders@uantwerpen.be (R.C.); 2 Disease Control Coordination, State Health Department, São Paulo 01246000, Brazil; 3 Institute of Tropical Pathology and Public Health, Federal University of Goiás, Goiânia 74605050, Brazil; edlaine@alumni.usp.br; 4 European AIDS Treatment Group, 1000 Brussels, Belgium; pieter.vanholder@eatg.org; 5 Sensoa, Flemish Expertise Centre for Sexual Health, 2060 Antwerp, Belgium; patrick.reyntiens@sensoa.be

**Keywords:** persons living with HIV, HIV care, COVID-19, PHQ-2, GAD-2, vaccination

## Abstract

COVID-19 affects persons living with HIV (PLWH) both directly (via morbidity/mortality) and indirectly (via disruption of HIV care). From July–November 2020, an online survey was conducted to investigate the psychosocial well-being of PLWH and changes in HIV care during the second semester of the COVID-19 outbreak. Data were collected on the socio-demographic characteristics of PLWH, their psychosocial well-being, impact of COVID-19 preventive measures on their daily routines and HIV follow-up. Of the 247 responses analyzed (mean age: 44.5 ± 13.2 years; 73.7% male), 67 (27.1%) and 69 (27.9%) respondents screened positive for anxiety (GAD-2 score ≥ 3) and depression (PHQ-2 score ≥ 3), respectively. HIV care had returned to pre-COVID-19 state for 48.6% PLWH, and 108 (43.7%) had no HIV follow-up during the past month. Over three quarters (76.1%) of respondents expressed willingness to receive the COVID-19 vaccine. Compared to previous findings in April 2020, substance use increased from 58.6% to 67.2% (*p* < 0.001). Our findings suggest that the well-being and medical follow-up of PLWH are still affected after almost a year into the COVID-19 outbreak. Remote HIV follow-up (telemedicine) with psychosocial support should be envisaged in the medium to long-term. Given that most PLWH accept COVID-19 vaccination, they may be prioritized for this intervention.

## 1. Introduction

December 2020 marked one year since the first case of coronavirus disease 2019 (COVID-19) was reported in China. Since then, over 80 million persons were confirmed to have been infected with the deadly virus, and more than 1.7 million COVID-19-related deaths had been reported around the globe by 31 December 2020 [1]. It is still unclear whether infection with HIV may affect the risk for, and outcomes of COVID-19. It may be that low CD4 counts in HIV-infected individuals spare them from developing the cytokine storm responsible for COVID-19 morbidity and-mortality [2], and it is equally speculated that some antiretroviral drugs may hinder coronavirus replication [3]. On the other hand, an immuno-depressed terrain may also create room for more severe clinical manifestations if infected with the novel coronavirus. Most of the previously available data did not provide evidence that persons living with HIV (PLWH) have a higher COVID-19 infection rate or a different disease course compared to those without HIV infection [4,5,6]. However, more recent studies suggest that COVID-19 infection in PLWH is associated with increased mortality and severe clinical outcomes particularly for those with CD4 counts below 200 cells/mm³ [7,8,9].

Besides causing disease and death in infected individuals, the COVID-19 outbreak has also disrupted healthcare provision for several non-COVID-19 conditions, including the routine follow-up of PLWH [10]. Moreover, the COVID-19 pandemic has resulted in a shrinking of funding for the global HIV response [11], and several countries reported high risks of stock-outs of antiretroviral treatment (ART) as a direct consequence of restrictions implemented to contain the COVID-19 outbreak [12]. Moreover, widespread inertia to visiting hospitals for non-COVID-19 health issues has been reported earlier during the pandemic [13], and it is unclear whether these tendencies still persist after several months. During an initial multi-country online survey in April 2020 [10], we found that 17.7% of respondents had difficulties in obtaining ART because of COVID-19–related measures. In addition, one quarter of participants were screened positive for either anxiety or depression. At the time, the global COVID-19 incidence was evolving exponentially, and most health systems were overwhelmed with COVID-19 cases. HIV clinics had to devise innovative ways to cater for PLWH amidst the challenges [10]. The purpose of this follow-up survey is to assess the psychosocial well-being of PLWH and the quality of HIV care offered to PLWH during the weeks and months following the first wave of COVID-19 infections.

## 2. Methods

### 2.1. Study Setting and Procedures

We conducted a multi-country, cross-sectional online survey among PLWH between 20 July and 30 November 2020. The study procedures were as follows: a web-based questionnaire was designed in English, translated to five other languages (Dutch, French, Portuguese, Spanish, and Russian), and hosted on the secure online platform of the International Citizen Project COVID-19 (ICPcovid) [14]. Only the English version (template questionnaire) had been pretested by our research team. Translations were also tested by local investigators prior to dissemination. The English version of the questionnaire is available as Supplementary Materials File S1.

The survey link was then disseminated widely via social media platforms as well as networks of various associations that support PLWH including the European AIDS Treatment Group (EATG), Sensoa (Flemish center of expertise for sexual health), RNP+Brasil (National Network of PLWH in Brazil), and RedLA+ (Latin American Network of Persons with HIV). Once potential participants clicked on the survey link, they were directed to a webpage on the ICPCovid website where they were given clear information about the study. Consenting participants were then able to access the online questionnaire and submit their responses. Respondents were equally encouraged to share the questionnaire with other PLWH in their network, in a snowball sampling approach. Questions were asked about socio-demographics, participation in the first online survey on HIV/COVID-19, and whether confinement measures were implemented (or lifted) in the place of residence of the respondent. In addition, we screened PLWH for psychosocial distress using the Patient Health Questionnaire (PHQ-2) [15] for depression and the Generalized Anxiety Disorder (GAD-2) tool [16] for anxiety. For both tools, a score of 3 and above was considered as positive screening. Of note, previous research had demonstrated the invariance of both PHQ and GAD scales across sex, strata and linguistic backgrounds [17]. Lastly, information was obtained regarding HIV follow-up, access to ART, and history of flu-like symptoms (only those symptoms listed as part of the World Health Organization’s clinical case definition for suspected COVID-19: fever, cough, general weakness/fatigue, headache, myalgia, sore throat, coryza, dyspnea, anorexia/nausea/vomiting, diarrhea) [18]. No participant names were collected, and the IP addresses of respondents were not tracked; the password-protected database was hosted online on the secure ICPcovid server in Belgium, accessible only to the principal investigators. Only respondents older than 18 years who self-confirmed being PLWH were included in our analysis.

### 2.2. Statistical Analysis

Continuous variables were reported as mean with standard deviation (mean ± SD) and compared across groups using non-parametric tests. Categorical variables were summarized as frequencies with percentages. Proportions were compared using the Chi-Squared test. A multiple logistic regression analysis with generalized estimating equations (GEE) was performed to investigate factors associated with visiting a health facility for routine HIV follow-up during the last month. Covariates for the final model were purposefully selected and included: socio-demographic variables (age, male/female sex, educational level, country classification as low and middle income countries (LMIC) vs. high income countries (HIC) based on the World Bank data [19]), psychosocial variables (PHQ-2 score, GAD-2 score, fear of getting infected with COVID-19 at a hospital (Likert score), presence/absence of psychosocial support at HIV clinic), history of lockdown, and history of flu vaccination during the last 12 months. The GEE were inserted into the model to control for correlations among respondents from the same country; in that respect, the cluster variable used was “country of residence.” Only countries with >10 responses were included in the multivariable model. Given the rapid and dynamic evolution of COVID-19, we included the “month of responding to the survey” as a covariate in the model to control for the influence of timing on the responses. Statistical analyses were performed in R version 4.0.2 (R Foundation for Statistical Computing, Vienna, Austria); the logistic regression model with GEE was constructed using the *geeglm* function in the R-package ‘Geepack’ [20]. All statistical tests were two-sided; *p* < 0.05 was considered statistically significant, and 95% confidence intervals (CI) were computed.

### 2.3. Ethical Considerations

The study protocol was approved by the Ethics Committee of the University of Antwerp (Belgium), Ref: 20/14/179. An e-consent was required before submitting the responses, and all data was submitted anonymously. The databases ICPcovid website were all compliant with the General Data Protection Regulation (GDPR) such that all collected information were treated with absolute confidentiality.

## 3. Results

### 3.1. Participants’ Characteristics

Overall, 264 responses were received but 17 were excluded in the analyses because the respondents were either younger than 18 years or did not confirm being PLWH. Therefore only 247 respondents were included in the analysis, their mean age was 44.5 ± 13.2 years, and 73.7% of them were males (Table 1). Responses originated from 26 countries, with a majority of respondents residing in Brazil (*n* = 83) and Belgium (*n* = 82) (Supplementary Materials File S2).

### 3.2. Psychosocial Well-Being

**Figure 1:**
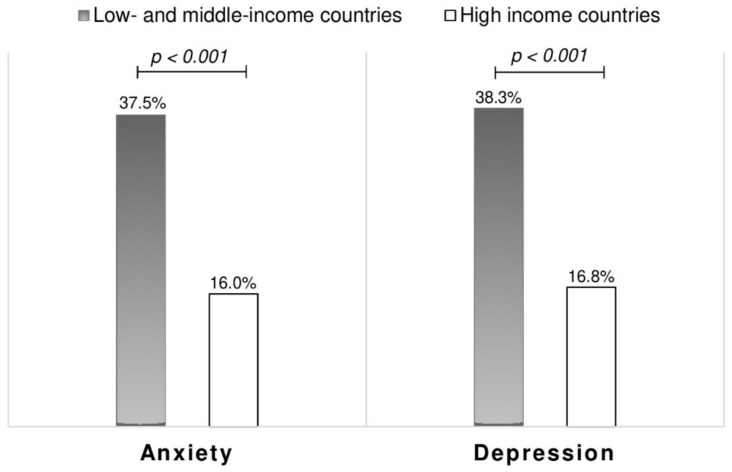
Prevalence of anxiety and depression among respondents.

Sixty-seven (27.1%) and 69 (27.9%) of respondents screened positive for anxiety and depression respectively. Forty-nine (19.8%) respondents had both anxiety and depression. Psychosocial distress was significantly higher among LMIC residents compared to HIC residents; *p* < 0.001 (Figure 1). Compared to males, female PLWH showed a higher prevalence of anxiety (37.7% vs. 23.6%; *p* = 0.048) and depression, although not statistically significant (37.7% vs. 24.7%; *p* = 0.074). Regional disparities in the burden of psychosocial problems wereobserved, with PLWH in Latin American countries reporting the highest prevalence of both anxiety and depression (38.8% and 40.0% respectively), followed by Eastern European countries (anxiety: 34.1%% and depression: 34.1%%) and Western Europe (anxiety: 15.5% and depression: 16.4%); *p* = 0.001. Comparing the four most represented countries (Belgium, Brazil, France, Russia), the prevalence of both anxiety and depression were highest in Brazil (respectively 39.8% and 41.0%), followed by Russia (respectively 32.1% and 25.0%), then Belgium (respectively 17.1% and 19.5%) and France (respectively 14.3% and 7.1%); Supplementary Materials File S3.

### 3.3. Flu-Like Symptoms

**Figure 2:**
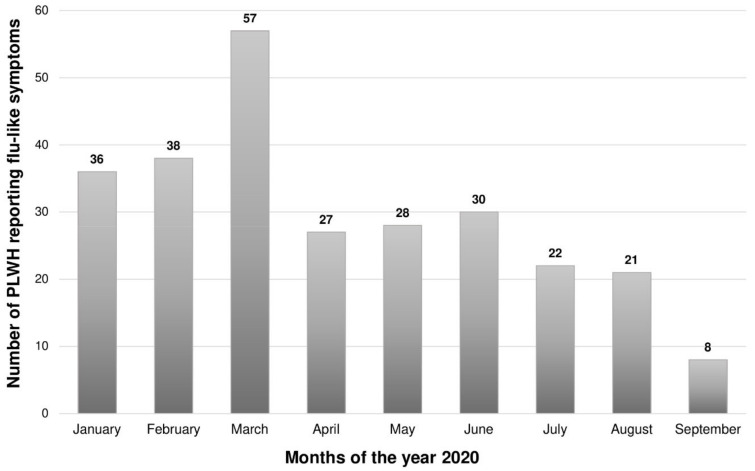
Frequency of flu-like symptoms among participants during the year 2020.

A total of 156 (63.2%) participants reported experiencing at least one flu-like symptom since the month of January 2020. Of note, data on the frequency of symptoms in October and November were not available due to involuntary omissions when designing the online questionnaire. The peak frequency of flu-like symptoms occurred in the month of March; see Figure 2. Only 6/156 (3.8%) of those with symptoms were hospitalized for severe illness. Moreover, 61 (24.7%) participants reported experiencing more than one episode of flu-like illness since January 2020.

### 3.4. COVID-19 Preventive Measures and Impact on Respondents

At the time of the survey, the respondents reported adopting the following measures for COVID-19 prevention: 206 (83.4%) wore face masks, 199 (80.6%) observed physical distancing >1 m, 199 (80.6%) regularly washed their hands while 172 (69.6%) used hand gels. While 175 (70.9%) participants reported that they had been vaccinated against flu during the past 12 months, 198/229 (86.5%) of them expressed willingness to receive the flu vaccine during the next flu season. On the question of COVID-19 vaccination, 188 (76.1%) of PLWH were willing to be vaccinated against COVID-19, 21 (8.5%) were against vaccination, while another 38 (15.4%) had no opinion. Excluding all PLWH with no opinion on COVID-19 vaccination, there was no significant difference in the proportion of vaccine-compliant respondents from LMIC (87.2%) and HIC (93.5%); *p* = 0.203. Lockdown measures had been implemented in the majority of localities where the participants resided, being reported by 180 (72.9%) respondents. However, only 154/180 (85.6%) of the latter thought that lockdown was necessary to control COVID-19 in their locality.

Regarding the impact of the pandemic on the lives of PLWH, nine (3.6%) respondents reported that they were unable to refill their ART during the past month, and only 109 (44.2%) were able to meet their HIV physician face-to-face during the past month. An additional 30 PLWH (12.1%) had benefited from teleconsultations, two third of whom had attained university level education. This implies that overall, 108 respondents (43.7%) had no form of follow-up during the past month; 65 of the latter resided in LMIC (representing 50.7% of LMIC respondents) while 43 were from HIC (representing 36.1% of HIC respondents); *p* = 0.021. Recent recreational substance use was reported by 119 (48.2%) of participants, including 84 (70.6%) consuming alcohol, 62 (52.1%) consuming tobacco, 29 (24.4%) marijuana, and 19 (16.0%) cocaine. The mean Likert score evaluating the level of fear/worry of being infected by COVID-19 during a hospital visit was 2.4 ± 1.2 (on a scale from 1 to 5); this fear was higher among participants from LMIC (mean Likert score: 2.73 ± 1.29) compared to those in HIC (mean Likert score: 2.03 ± 1.03); *p* < 0.001. Table 2 summarizes the self-reported difficulties caused by the implementation of COVID-19 restrictions, as well as difficulties encountered to get back to normal life (as before the pandemic) when restrictive measures were relaxed. Since there were several missing values in this section of the questionnaire, the denominators may differ from one row to the other.

Following the COVID-19 outbreak and associated measures, up to 99 (40.1%) PLWH mentioned that they needed psychosocial support, and the majority of participants (68.0%) acknowledged that some form of psychosocial support was available at their follow-up clinic. Of note, psychosocial support was more available for PLWH residing in HIC (94/119; 79.0%) compared to those in LMIC (74/128; 57.8%); *p* = 0.001. Almost half of the respondents (120/247; 48.6%) reported that HIV care at the healthcare facility had returned to the way it was before the COVID-19 pandemic.

### 3.5. Factors Associated with Visiting a Health Facility for HIV Follow-Up

The multivariable model revealed that more educated persons and those residing in LMIC had lower odds of having visited a hospital/clinic for routine HIV follow-up during the past month (Table 3).

### 3.6. Cohort of PLWH Who Were Identifiable in Previous (April 2020) and Current (July 2020) Surveys

Using encrypted email addresses, we were able to link the responses from both surveys for 58 respondents (mean age 44.7 ± 13.1 years; 75.9% male; 17.2% with GAD-2 scores ≥ 3; and 22.4% with PHQ-2 scores ≥ 3). Majority of PLWH in the cohort resided in Brazil (43.1%) and Belgium (29.3%). There was an overall non-significant increase in both GAD-2 scores (from 1.74 ± 1.53 to 1.91 ± 1.65) and PHQ-2 scores (from 1.66 ± 1.56 to 1.88 ± 1.52) between the first and second surveys; paired Wilcoxon signed rank test *p*-values of 0.290 and 0.461, respectively. However, the proportion of respondents who reported using recreational drugs in this cohort significantly increased from 58.6% during the initial survey to 67.2% in the follow-up survey; *p* < 0.001. Regarding COVID-19 preventive measures, face mask usage markedly increased from 60.3% to 91.4% (*p* < 0.001), while observance of physical distancing was fairly similar across surveys (96.6% vs. 93.1%; *p* = 0.402). The proportion of PLWH in our cohort who reported visiting the HIV clinic during the past month increased from 24.1% to 44.8% for the initial and follow-up survey, respectively (*p* = 0.019).

## 4. Discussion

Our study throws more light on the well-being of PLWH as well as the state of HIV care in the context of a persisting pandemic that has greatly impacted health systems. A little over one quarter of respondents screened positive for either anxiety or depression, representing a fairly stable prevalence compared to the 22% in a first survey among PLWH earlier during the pandemic [10]. Compared to anxiety and depression rates among PLWH prior to the COVID-19 outbreak [21,22], our findings show a slightly lower prevalence of both conditions. However, differences in the psychometric tools used for screening render comparisons across studies difficult. In addition, the online approach of our survey may have introduced a selection bias by recruiting mostly PLWH in relatively good psychosocial health and who were motivated to fill the questionnaire. Of note, paired analysis on a small cohort of respondents (having very similar socio-demographic characteristics as the entire study population) showed a trend of increasing PHQ-2 and GAD-2 scores over time, albeit being non-significant statistically. One reason for this observation could be increasing infection and death toll due to COVID-19 around the world, causing millions around the world to become more and more concerned about their health. A similar trend of increasing anxiety and depression during the COVID-19 pandemic was noted in the Belgian general population [23].

We sought to compare our findings with data from the general population within the same country. Although all the surveys from the general population available in literature employed the PHQ-9 and GAD-7 scales, these have been shown to yield similar estimates as the PHQ-2 and GAD-2 scales which we used for screening depression and anxiety respectively [24,25]. Compared to PLWH (our study), the frequency of depression and anxiety among the general population during the COVID-19 pandemic was similar in Belgium [23] but higher in Brazil [26], France [27], and Russia [28]. These data are encouraging as they suggest that the pandemic’s toll on psychosocial well-being is not more severe among PLWH compared to the general population.

In line with pre-COVID-19 reports suggesting an overall greater burden of depression among PLWH in developing nations [21], we observed in this study that both anxiety and depression were significantly more prevalent among PLWH from LMIC. This same trend was already observed during our first online survey among PLWH [10]. An explanation could be that the COVID-19 outbreak has been more detrimental to the already weak health systems in LMIC. Indeed, more PLWH in LMIC were deprived of HIV care during the month preceding the survey compared to those in HIC, and residing in LMIC was associated with reduced odds of having visited the HIV clinic during the past month. Furthermore, the fear of getting infected with COVID-19 was significantly higher in LMIC than in HIC. All these setbacks, amongst others, would further jeopardize HIV care and access to ART [12] thereby generating more distress and uncertainties among PLWH in LMIC. The negative impacts of the COVID-19 pandemic on HIV care pose a serious threat to the great progress achieved thus far in the four-decade fight against HIV/AIDS [29]. In order to sustainably gain grounds in the global HIV response, it is crucial that healthcare systems improve the provision of HIV-related services during and after the COVID-19 pandemic [30], and that PLWH be continuously sensitized to ensure optimal follow-up during this period.

We observed that about one third of respondents had reported a worsening of their financial status during the pandemic. While we are unable to confirm if these changes were directly related to COVID-19 or associated preventive measures, it is obvious that nationwide lockdowns had their toll on the socio-economic situation of several countries [31]. In addition, over half of the respondents found it difficult to return to their ‘normal’ sexual and social lives even after the restrictions were relaxed. This finding underscores the possibility that the punctual psychosocial distress brought about by the COVID-19 outbreak may have medium to long-term consequences on PLWH, requiring that they be carefully monitored and counselled over a prolonged period of time. Although 68% of respondents mentioned the availability of psychosocial support at their HIV clinics, there is a need to scale up and sustain this system particularly in LMIC.

A notable finding in this study is that while only 70% of the surveyed PLWH admitted receiving a flu vaccine in the past year, more than 85% of them were willing to be vaccinated against flu in the near future. Not only is flu vaccination encouraged among PLWH worldwide, but it will also prevent them from developing flu-symptoms which may mimic COVID-19 and become an additional concern for both PLWH and healthcare providers [10]. A similar enthusiasm was noted regarding COVID-19 vaccination. The 15.4% PLWH who were still undecided about whether they wanted to be vaccinated with a COVID-19 vaccine may be explained by the fact that the survey was closed before the first COVID-19 vaccines were officially approved and deployed. Reasons for vaccine hesitancy among a minority of PLWH need to be further investigated.

In our multivariable analysis, we found that more educated participants were less likely to have visited a health facility for HIV follow-up during the past month. The fact that most of the respondents who resorted to teleconsultations were university graduates suggests that the more literate PLWH had adapted to the COVID-19 restrictions by finding ways to be followed up without necessarily going to the hospital. Telemedicine could indeed prove very useful during health crises like the COVID-19 pandemic to remotely follow-up persons with chronic conditions such as HIV/AIDS [10,32]. The only covariate that was positively associated with visiting the HIV clinic during the past month was participation in the survey in the month of September (which implies that the responding PLWH visited the clinic in August). By August, most countries had relaxed their COVID-19 restrictions thereby allowing PLWH to visit the HIV clinic with greater ease. However, with the progressive advent of a second wave towards the end of summer, stricter preventive measures were implemented most likely resulting in more difficulties to conduct face-to-face follow-up of PLWH. One possible consequence of visiting hospitals less frequently during the COVID-19 outbreak is the frequent clinical deterioration of patients during the second half of 2020, as evidenced by an increased number of PLWH being admitted with advanced HIV/AIDS, lower CD4 counts and higher viral loads [33]. All these elements further highlight the need to implement innovative yet effective methods for remote HIV care.

In the multivariable model, none of the psychosocial variables (PHQ-2 scores, GAD-2 scores, fear of getting infected with COVID-19, psychosocial support at HIV clinic) significantly altered the odds of having visited the HIV clinic during the past month. This suggests that at the time of this survey, PLWH were not routinely attending HIV clinics every month regardless of their degree of psychological distress. Indeed, more than half of respondents reported that HIV care had not yet returned to the way it was prior to the COVID-19 outbreak.

Several limitations must be mentioned regarding the current study. This was a cross-sectional survey with a small, nested cohort and very limited sample sizes for some countries, making it difficult to conclude on the exact role of the COVID-19 pandemic in causing the reported observations. In addition, our sampling approach was not ideal and may have recruited PLWH with biased socio-demographic, clinical, psychosocial profiles since only certain individuals (relatively healthy PLWH who had access to good internet) were able to participate. Adding to the selectivity of the web-based approach used in this survey, the snowball recruitment method also introduces substantial selection bias; indeed, the first participants would greatly influence the remaining participants as they would share the questionnaire to their own networks. This was partially mitigated by sharing the survey link on several different platforms. Thus, our findings are hardly generalizable to all PLWH. Furthermore, the veracity of the information provided by the respondents cannot be verified by the research team, and there were some missing values in the responses. We also acknowledge that our overall convenience-based sample size may be small, and that the four-month study duration amidst a dynamic and rapidly evolving pandemic may lead to highly variable findings across different time points and different countries, justifying the statistical techniques employed in our multivariable analysis.

In conclusion, one year after the identification of the first COVID-19 case, the psychosocial well-being of, and provision of care to PLWH is still affected. Means of ensuring remote follow-up and good psychosocial support should urgently be deployed to stall the increasing levels of anxiety and depression among PLWH during the ongoing pandemic, but also in the medium to long-term. The advent of an effective COVID-19 vaccine may put PLWH at ease by reducing their risk of becoming infected. Given that PLWH may be at increased risk for developing more severe COVID-19 disease and considering the detrimental implications of the pandemic on their psychosocial well-being [34], PLWH should be considered as a priority population for COVID-19 vaccination. This should be feasible in both LMIC and HIC settings since most PLWH are willing to be vaccinated against the deadly virus.

## Figures and Tables

**Table 1:** Characteristics of study participants.

Characteristics	Observed
Age, years: mean (SD)	44.5 (13.2)
**Sex: *n* (%)**	
Male	182 (73.7%)
Female	61 (24.7%)
Other	4 (1.6%)
**Classification of country of residence: *n* (%)**	
Low and Middle income country (LMIC)	128 (51.8%)
High income country (HIC)	119 (48.2%)
**Highest education level: *n* (%)**	
Primary	19 (7.7%)
Secondary	73 (29.6%)
Undergraduate	75 (30.4%)
Post-graduate	80 (32.4%)
**Religion: *n* (%)**	
Christian	123 (49.8%)
Muslim	6 (2.4%)
Other	31 (12.6%)
None	87 (35.2%)
**Marital status: *n* (%)**	
Single	101 (40.9%)
Stable relationship	34 (13.8%)
Cohabitation	33 (13.4%)
Married	48 (19.4%)
Divorced	23 (9.3%)
Other	8 (3.2%)
Visited health facility or HIV clinic during the last month for routine HIV follow-up: n (%)	132 (53.4%)
**COVID-19 test results: *n* (%)**	
Positive	9/59 (15.3%)
Negative	50/59 (84.7%)

**Table 2:** Impact of the COVID-19 pandemic and associated restrictions on the lives of PLWH.

Reported Changes during the Confinement Period: *n* (%)				
	No Change	Better	Worse	Missing Data
Financial situation (*n* = 175)	97 (55.4%)	13 (7.4%)	65 (37.1%)	72
Sexual fulfilment (*n* = 160)	73 (45.6%)	11 (6.9%)	76 (47.5%)	87
Family life (*n* = 166)	82 (49.4%)	21 (12.7%)	63 (38.0%)	81
Social life outside family (*n* = 173)	48 (27.7%)	10 (5.8%)	115 (66.5%)	74
**Reported changes after relaxing confinement measures: *n* (%)**				
		**Yes**	**No**	**Missing data**
Difficulty to revert to normal social life (*n* = 183)		91 (49.7%)	92 (50.3%)	64
Difficulty to revert to normal sexual life (*n* = 183)		71 (38.8%)	112 (61.2%)	64

**Table 3:** Multiple logistic regression investigating factors associated with visiting a health facility for HIV follow-up during the past month (*n* = 204).

Covariates	Adjusted OR (95% CI)	*p* Value
Age (in years)	0.984 (0.967–1.00)	0.086
Sex		
Female	Reference	
Male	0.915 (0.766–1.09)	0.326
Educational level		
Primary	Reference	
Secondary	0.532 (0.310–0.911)	0.022
Undergraduate	0.856 (0.223–3.29)	0.821
Postgraduate	0.247 (0.090–0.683)	0.007
Country classification		
HIC	Reference	
LMIC	0.368 (0.331–0.409)	<0.001
PHQ-2 score	0.907 (0.788–1.04)	0.172
GAD-2 score	1.09 (0.856–1.39)	0.479
Fear of getting infected at hospital (Likert score)	1.07 (0.948–1.22)	0.261
Availability of psychosocial support at HIV clinic	0.837 (0.305–2.30)	0.73
Flu vaccination during the past 12 months	1.10 (0.535–2.26)	0.797
History of lockdown in respondent’s locality	1.20 (0.649–2.23)	0.558
Month of participation in online survey		
July	Reference	
August	2.11 (0.716–6.20)	0.176
September	3.31 (1.47–7.43)	0.004
October	0.636 (0.010–39.5)	0.83
November	0.785 (0.437–1.41)	0.419

OR: Odds Ratio. CI: Confidence Interval.

## Data Availability

The data presented in this paper are available upon reasonable request to the corresponding author.

## Supplementary Materials

The following are available online at https://www.mdpi.com/article/10.3390/ijerph18094635/s1. Supplementary File S1 (English Questionnaire for the online survey); Supplementary File S2 (Participating countries and number of respondents); Supplementary File S3 (Comparison of respondents from the four most represented countries).
