# Supplementary material for: Follow-Up Survey of the Impact of COVID-19 on People Living with HIV during the Second Semester of the Pandemic
Source: Int J Environ Res Public Health. 2021 Apr 27;18(9):4635. doi: 10.3390/ijerph18094635 (PMC8123847; doi:10.3390/ijerph18094635)
Supplement: Supplementary file 1 [file ijerph-18-04635-s001.zip › Supplementary S1.pdf]

Covid-HIV ROUND 2

General questions

INTRODUCTION

As the Covid-19 containment measures ramp up around the world, different countries implement different strategies, and health systems are overwhelmed to varying degrees. Generating high-quality evidence on the impact of Covid-19 and related measures on both the quality of life and the management of HIV in different settings will help provide guidance for decision-making and better preparedness in case of future pandemics. In this light, researchers based at the University of Antwerp (Belgium) have designed a small study in collaboration with Sensoa and EATG to investigate the impact of the Covid-19 pandemic on HIV care and the well-being of persons living with HIV (PLHIV).

We kindly ask PLHIV to voluntarily fill this brief survey (about 10 minutes). The requested information is totally anonymous, and your answers will contribute to a better understanding how PLHIV are affected by the ongoing Covid-19 outbreak.

NB: (*) identifies obligatory questions

Q: I am a person living with HIV (*)

Type: choice

A: one of the following:

yes	=>	*Yes*
no	=>	*No*

Q: Age (in years) (*)

Type: number

A: number (min: 1 / max: 110 / step: 1)

Q: Gender (*)

Type: choice

A: one of the following:

male	=>	*Male*
female	=>	*Female*
non_binary	=>	*Non-binary*
other	=>	*Other*

Q: Which country do you currently live in? (*)

Type: choice

A: one of the following:

Afghanistan	=>	*Afghanistan*
Albania	=>	*Albania*
Algeria	=>	*Algeria*
American Samoa	=>	*American Samoa*
Andorra	=>	*Andorra*
Angola	=>	*Angola*

Anguilla	=>	*Anguilla*
Antarctica	=>	*Antarctica*
Antigua & Barbuda	=>	*Antigua & Barbuda*
Argentina	=>	*Argentina*
Armenia	=>	*Armenia*
Aruba	=>	*Aruba*
Ascension Island	=>	*Ascension Island*
Australia	=>	*Australia*
Austria	=>	*Austria*
Azerbaijan	=>	*Azerbaijan*
Bahamas	=>	*Bahamas*
Bahrain	=>	*Bahrain*
Bangladesh	=>	*Bangladesh*
Barbados	=>	*Barbados*
Belarus	=>	*Belarus*
Belgium	=>	*Belgium*
Belize	=>	*Belize*
Benin	=>	*Benin*
Bermuda	=>	*Bermuda*
Bhutan	=>	*Bhutan*
Bolivia	=>	*Bolivia*
Bosnia & Herzegovina	=>	*Bosnia & Herzegovina*
Botswana	=>	*Botswana*
Bouvet Island	=>	*Bouvet Island*
Brazil	=>	*Brazil*
British Indian Ocean Territory	=>	*British Indian Ocean Territory*
British Virgin Islands	=>	*British Virgin Islands*
Brunei	=>	*Brunei*
Bulgaria	=>	*Bulgaria*
Burkina Faso	=>	*Burkina Faso*
Burundi	=>	*Burundi*
Cambodia	=>	*Cambodia*
Cameroon	=>	*Cameroon*
Canada	=>	*Canada*
Canary Islands	=>	*Canary Islands*
Cape Verde	=>	*Cape Verde*
Caribbean Netherlands	=>	*Caribbean Netherlands*
Cayman Islands	=>	*Cayman Islands*
Central African Republic	=>	*Central African Republic*
Ceuta & Melilla	=>	*Ceuta & Melilla*
Chad	=>	*Chad*
Chile	=>	*Chile*
China	=>	*China*
Christmas Island	=>	*Christmas Island*
Clipperton Island	=>	*Clipperton Island*
Cocos (Keeling) Islands	=>	*Cocos (Keeling) Islands*

Colombia	=>	*Colombia*
Comoros	=>	*Comoros*
Congo - Brazzaville	=>	*Congo - Brazzaville*
Congo - Kinshasa	=>	*Congo - Kinshasa*
Cook Islands	=>	*Cook Islands*
Costa Rica	=>	*Costa Rica*
Croatia	=>	*Croatia*
Cuba	=>	*Cuba*
Curaçao	=>	*Curaçao*
Cyprus	=>	*Cyprus*
Czechia	=>	*Czechia*
Côte d’Ivoire	=>	*Côte d’Ivoire*
Denmark	=>	*Denmark*
Diego Garcia	=>	*Diego Garcia*
Djibouti	=>	*Djibouti*
Dominica	=>	*Dominica*
Dominican Republic	=>	*Dominican Republic*
Ecuador	=>	*Ecuador*
Egypt	=>	*Egypt*
El Salvador	=>	*El Salvador*
Equatorial Guinea	=>	*Equatorial Guinea*
Eritrea	=>	*Eritrea*
Estonia	=>	*Estonia*
Eswatini	=>	*Eswatini*
Ethiopia	=>	*Ethiopia*
Falkland Islands	=>	*Falkland Islands*
Faroe Islands	=>	*Faroe Islands*
Fiji	=>	*Fiji*
Finland	=>	*Finland*
France	=>	*France*
French Guiana	=>	*French Guiana*
French Polynesia	=>	*French Polynesia*
French Southern Territories	=>	*French Southern Territories*
Gabon	=>	*Gabon*
Gambia	=>	*Gambia*
Georgia	=>	*Georgia*
Germany	=>	*Germany*
Ghana	=>	*Ghana*
Gibraltar	=>	*Gibraltar*
Greece	=>	*Greece*
Greenland	=>	*Greenland*
Grenada	=>	*Grenada*
Guadeloupe	=>	*Guadeloupe*
Guam	=>	*Guam*
Guatemala	=>	*Guatemala*
Guernsey	=>	*Guernsey*

Guinea	=>	*Guinea*
Guinea-Bissau	=>	*Guinea-Bissau*
Guyana	=>	*Guyana*
Haiti	=>	*Haiti*
Heard & McDonald Islands	=>	*Heard & McDonald Islands*
Honduras	=>	*Honduras*
Hong Kong SAR China	=>	*Hong Kong SAR China*
Hungary	=>	*Hungary*
Iceland	=>	*Iceland*
India	=>	*India*
Indonesia	=>	*Indonesia*
Iran	=>	*Iran*
Iraq	=>	*Iraq*
Ireland	=>	*Ireland*
Isle of Man	=>	*Isle of Man*
Israel	=>	*Israel*
Italy	=>	*Italy*
Jamaica	=>	*Jamaica*
Japan	=>	*Japan*
Jersey	=>	*Jersey*
Jordan	=>	*Jordan*
Kazakhstan	=>	*Kazakhstan*
Kenya	=>	*Kenya*
Kiribati	=>	*Kiribati*
Kosovo	=>	*Kosovo*
Kuwait	=>	*Kuwait*
Kyrgyzstan	=>	*Kyrgyzstan*
Laos	=>	*Laos*
Latvia	=>	*Latvia*
Lebanon	=>	*Lebanon*
Lesotho	=>	*Lesotho*
Liberia	=>	*Liberia*
Libya	=>	*Libya*
Liechtenstein	=>	*Liechtenstein*
Lithuania	=>	*Lithuania*
Luxembourg	=>	*Luxembourg*
Macao SAR China	=>	*Macao SAR China*
Madagascar	=>	*Madagascar*
Malawi	=>	*Malawi*
Malaysia	=>	*Malaysia*
Maldives	=>	*Maldives*
Mali	=>	*Mali*
Malta	=>	*Malta*
Marshall Islands	=>	*Marshall Islands*
Martinique	=>	*Martinique*
Mauritania	=>	*Mauritania*

Mauritius	=>	*Mauritius*
Mayotte	=>	*Mayotte*
Mexico	=>	*Mexico*
Micronesia	=>	*Micronesia*
Moldova	=>	*Moldova*
Monaco	=>	*Monaco*
Mongolia	=>	*Mongolia*
Montenegro	=>	*Montenegro*
Montserrat	=>	*Montserrat*
Morocco	=>	*Morocco*
Mozambique	=>	*Mozambique*
Myanmar (Burma)	=>	*Myanmar (Burma)*
Namibia	=>	*Namibia*
Nauru	=>	*Nauru*
Nepal	=>	*Nepal*
Netherlands	=>	*Netherlands*
Netherlands Antilles	=>	*Netherlands Antilles*
New Caledonia	=>	*New Caledonia*
New Zealand	=>	*New Zealand*
Nicaragua	=>	*Nicaragua*
Niger	=>	*Niger*
Nigeria	=>	*Nigeria*
Niue	=>	*Niue*
Norfolk Island	=>	*Norfolk Island*
Northern Mariana Islands	=>	*Northern Mariana Islands*
North Korea	=>	*North Korea*
North Macedonia	=>	*North Macedonia*
Norway	=>	*Norway*
Oman	=>	*Oman*
Outlying Oceania	=>	*Outlying Oceania*
Pakistan	=>	*Pakistan*
Palau	=>	*Palau*
Palestinian Territories	=>	*Palestinian Territories*
Panama	=>	*Panama*
Papua New Guinea	=>	*Papua New Guinea*
Paraguay	=>	*Paraguay*
Peru	=>	*Peru*
Philippines	=>	*Philippines*
Pitcairn Islands	=>	*Pitcairn Islands*
Poland	=>	*Poland*
Portugal	=>	*Portugal*
Puerto Rico	=>	*Puerto Rico*
Qatar	=>	*Qatar*
Romania	=>	*Romania*
Russia	=>	*Russia*
Rwanda	=>	*Rwanda*

Réunion	=>	*Réunion*
Samoa	=>	*Samoa*
San Marino	=>	*San Marino*
Saudi Arabia	=>	*Saudi Arabia*
Senegal	=>	*Senegal*
Serbia	=>	*Serbia*
Seychelles	=>	*Seychelles*
Sierra Leone	=>	*Sierra Leone*
Singapore	=>	*Singapore*
Sint Maarten	=>	*Sint Maarten*
Slovakia	=>	*Slovakia*
Slovenia	=>	*Slovenia*
Solomon Islands	=>	*Solomon Islands*
Somalia	=>	*Somalia*
South Africa	=>	*South Africa*
South Georgia & South Sandwich Islands	=>	*South Georgia & South Sandwich Islands*
South Korea	=>	*South Korea*
South Sudan	=>	*South Sudan*
Spain	=>	*Spain*
Sri Lanka	=>	*Sri Lanka*
St. Barthélemy	=>	*St. Barthélemy*
St. Helena	=>	*St. Helena*
St. Kitts & Nevis	=>	*St. Kitts & Nevis*
St. Lucia	=>	*St. Lucia*
St. Martin	=>	*St. Martin*
St. Pierre & Miquelon	=>	*St. Pierre & Miquelon*
St. Vincent & Grenadines	=>	*St. Vincent & Grenadines*
Sudan	=>	*Sudan*
Suriname	=>	*Suriname*
Svalbard & Jan Mayen	=>	*Svalbard & Jan Mayen*
Sweden	=>	*Sweden*
Switzerland	=>	*Switzerland*
Syria	=>	*Syria*
São Tomé & Príncipe	=>	*São Tomé & Príncipe*
Taiwan	=>	*Taiwan*
Tajikistan	=>	*Tajikistan*
Tanzania	=>	*Tanzania*
Thailand	=>	*Thailand*
Timor-Leste	=>	*Timor-Leste*
Togo	=>	*Togo*
Tokelau	=>	*Tokelau*
Tonga	=>	*Tonga*
Trinidad & Tobago	=>	*Trinidad & Tobago*
Tristan da Cunha	=>	*Tristan da Cunha*
Tunisia	=>	*Tunisia*
Turkey	=>	*Turkey*

Turkmenistan	=>	*Turkmenistan*
Turks & Caicos Islands	=>	*Turks & Caicos Islands*
Tuvalu	=>	*Tuvalu*
U.S. Outlying Islands	=>	*U.S. Outlying Islands*
U.S. Virgin Islands	=>	*U.S. Virgin Islands*
Uganda	=>	*Uganda*
Ukraine	=>	*Ukraine*
United Arab Emirates	=>	*United Arab Emirates*
United Kingdom	=>	*United Kingdom*
United States	=>	*United States*
Uruguay	=>	*Uruguay*
Uzbekistan	=>	*Uzbekistan*
Vanuatu	=>	*Vanuatu*
Vatican City	=>	*Vatican City*
Venezuela	=>	*Venezuela*
Vietnam	=>	*Vietnam*
Wallis & Futuna	=>	*Wallis & Futuna*
Western Sahara	=>	*Western Sahara*
Yemen	=>	*Yemen*
Zambia	=>	*Zambia*
Zimbabwe	=>	*Zimbabwe*
Åland Islands	=>	*Åland Islands*

Q: Religion (*)

Type: choice

A: one of the following:

christian	=>	*Christian*
muslim	=>	*Muslim*
other	=>	*Other*
none	=>	*None*

Q: Highest educational level (*)

Type: choice

A: one of the following:

primary	=>	*Primary*
Secondary	=>	*Secondary*
University Undergraduate degree holder	=>	*University Undergraduate degree holder*
University Postgraduate degree holder	=>	*University Postgraduate degree holder*
none	=>	*None*

Q: Marital status (*)

Type: choice

A: one of the following:

single	=>	*Single*
legally_married	=>	*Legally married*
cohabitation	=>	*Cohabitation*
divorced	=>	*Divorced*
widow_widower	=>	*Widow/widower*
not_married_not_cohab_but_in_relationship	=>	*I am not married, nor in cohabitation, but I am in a relationship*
other	=>	*Other*

Q: Did you fill the previous ICPCovid questionnaire about COVID-19 and HIV on this website a few weeks ago?

Type: choice

A: one of the following:

yes	=>	*Yes, I filled the previous questionnaire*
no	=>	*No, this is my first time to fill the questionnaire*

General health issues

Q: Have you been vaccinated against flu during the past 12 months? (*)

Type: choice

A: one of the following:

yes	=>	*Yes*
no	=>	*No*

Q: Do you intend to take the flu vaccine during the next flu season?(*)

Type: choice

A: one of the following:

yes	=>	*Yes*
no	=>	*No*
dont_know	=>	*I don't know*

Q: Since the beginning of 2020, have you experienced any of the following symptoms? (multiple options possible)(*)

Type: choice_multiple

A: multiple answers possible:

fever	=>	*Fever*
headaches	=>	*Headaches*
sore_throat	=>	*Sore throat*
loss_taste	=>	*Loss of taste*
loss_smell	=>	*Loss of smell*
stuffy_nose	=>	*Stuffy and/or running nose*
dry_cough	=>	*Dry cough*

productive_cough	=>	*Productive cough*
shortness_breath	=>	*Shortness of breath*
muscle_pain	=>	*Muscle or body pains*
weakness	=>	*General weakness*
nausea	=>	*Nausea*
diarrhea	=>	*Diarrhea*
other	=>	*Other (specify)*
none	=>	*No symptom*

Q: Specify other symptom

Type: text

A: text input

Visible if

Q:	A:
:input[name="which_symptoms_did_you_experience_multiple_options_possible_[other]"]	- checked => 1

Q: In which month(s) did you experience flu-like symptoms? (many answers possible) (*)

Type: choice_multiple

A: multiple answers possible:

January	=>	*January 2020*
February	=>	*February 2020*
March	=>	*March 2020*
April	=>	*April 2020*
May	=>	*May 2020*
June	=>	*June 2020*
July	=>	*July 2020*
August	=>	*August 2020*
September	=>	*September 2020*

Visible if

Q:	A:
:input[name="which_symptoms_did_you_experience_multiple_options_possible_[none]"]	- unchecked => 1

Q: If you experienced any of these symptoms, were you hospitalised for this illness? (*)

Type: choice

A: one of the following:

yes	=>	*Yes*
no	=>	*No*

Visible if

Q:	A:
:input[name="which_symptoms_did_you_experience_multiple_options_possible_[none]"]	- unchecked => 1

Q: Were lockdown measures implemented for COVID-19 in the area where you live? (*)

Type: choice

A: one of the following:

yes	=>	*Yes*
no	=>	*No*

Q: If yes, how were the following aspects of your life affected by the lockdown?(*)

Type: choice_scale

A: My financesMy sexual lifeMy family lifeMy social life (with friends, neighbours, etc)

better	=>	*Became better*
worse	=>	*Became worse*
same	=>	*No change*
NA	=>	*Not applicable*

Visible if

Q:	A:
Were lockdown measures implemented for COVID-19 in the area where you live?	- value => yes

Q: During the last 2 weeks, how often have you experienced the following: (*)

Type: choice_scale

A: Little interest or pleasure in doing thingsFeeling down, depressed or hopelessFeeling nervous, anxious or on edgeNot being able to stop or control worrying

0	=>	*Not at all*
1	=>	*Several days*
2	=>	*More than half the days*
3	=>	*Nearly every day*

Q: Besides your treatment for HIV and other chronic diseases, did you start taking other medications during the COVID-19 epidemic period? (*)

Type: choice

A: one of the following:

yes	=>	*Yes*
no	=>	*No*

Q: If yes, which kind of drugs did you start taking? (many answers possible)(*)

Type: choice_multiple

A: multiple answers possible:

anti_depressant	=>	*Drugs against depression*
anxiolytics	=>	*Drugs against anxiety/restlessness*
anti_covid	=>	*Drugs to prevent COVID-19*
sexual	=>	*Drugs to improve sexual drive or performance*
boost_immunity	=>	*Drugs to strengthen my immune system*
anti_infective	=>	*Drugs to treat a new infection (bacteria, parasite, etc)*
other	=>	*Other drugs*

Visible if

Q:	A:
Besides your treatment for HIV and other chronic diseases, did you start taking other medications during the COVID-19 epidemic period?	- value => yes

Q: Specify other type of drugs

Type: text

A: text input

Visible if

Q:	A:
:input[name="if_yes_which_kind_of_drugs_did_you_take_many_answers_possible_[other]"]	- checked => 1

Q: Are you taking these drugs presently? (*)

Type: choice

A: one of the following:

yes	=>	*Yes*
no	=>	*No*

Visible if

Q:	A:
Besides your treatment for HIV and other chronic diseases, did you start taking other medications during the COVID-19 epidemic period?	- value => yes

Q: During the COVID-19 epidemic, have you been taking any of the recreational substances listed below? (multiple options possible) (*)

Type: choice_multiple

A: multiple answers possible:

alcohol	=>	*Alcohol*
tobacco	=>	*Tobacco*
vaping	=>	*Vaping*
cocaine	=>	*Cocaine*
heroine	=>	*Heroine*

marijuana	=>	*Marijuana/Cannabis*
Poppers	=>	*Poppers*
crystal_meth	=>	*Crystal meth*
amphetamines	=>	*Amphetamines / Methamphetamines*
GHB	=>	*GHB*
Ketamine	=>	*Ketamine*
other	=>	*Other*
none	=>	*No recreational substance use*

Q: Are you taking these recreational substances presently? (*)

Type: choice

A: one of the following:

yes	=>	*Yes*
no	=>	*No*

Visible if

Q:	A:
:input[name="during_the_lockdown_have_you_been_taking_any_of_the_recreational[none]"]	- unchecked => 1

COVID-19 Transmission and Prevention

Q: According to you, what is the COVID-19 situation now in the area where you live? (*)

Type: choice_scale

A: Number of new COVID-19 casesNumber of new COVID-19 deaths

increasing	=>	*Increasing as the days go by*
decreasing	=>	*Decreasing as the days go by*
constant	=>	*Constant, neither increasing nor decreasing*
dont_know	=>	*I don't know*
NA	=>	*Not applicable*

Q: Do you think that the lockdown measures were necessary to control COVID-19 in the town where you live? (*)

Type: choice

A: one of the following:

yes	=>	*Yes*
no	=>	*No*

Visible if

Q:	A:
Were lockdown measures implemented for COVID-19 in the area where you live?	- value => yes

Q: Have COVID-19 lockdown measures currently been relaxed in the area where you live? (*)

Type: choice

A: one of the following:

yes	=>	*Yes*
no	=>	*No*
na	=>	*Not applicable (there has never been lockdown)*

Q: Have you experienced any difficulties in getting back to social life after the COVID-19 restrictions were relaxed?(*)

Type: choice

A: one of the following:

yes	=>	*Yes*
no	=>	*No*

Visible if

Q:	A:
Have COVID-19 lockdown measures currently been relaxed in the area where you live?	- value => yes

Q: Have you experienced any difficulties in getting back to your normal sexual life after the COVID-19 restrictions were relaxed? (*)

Type: choice

A: one of the following:

yes	=>	*Yes*
no	=>	*No*

Visible if

Q:	A:
Have COVID-19 lockdown measures currently been relaxed in the area where you live?	- value => yes

Q: To what extent do you fear a possible new lockdown? (*)

Type: choice_scale

A: On a scale of 1 (no fear) to 5 (extreme fear)

1	=>	*1*
2	=>	*2*
3	=>	*3*
4	=>	*4*
5	=>	*5*

Visible if

Q:	A:
Have COVID-19 lockdown measures currently been relaxed in the area where you live?	- value => yes

Q: Which protective measure are you currently using to avoid coronavirus? (multiple options possible)(*)

Type: choice_multiple

A: multiple answers possible:

distancing	=>	*I keep a physical distance of at least 1-2 meters from others*
face_mask	=>	*I wear a face mask when going outside*
cough_tissue	=>	*When I cough or sneeze, I cover my mouth and nose with a paper tissue or with the crease of my elbow*
cough_wash_hands	=>	*When I cough or sneeze, I wash my hands shortly afterwards*
body_temp_every_week	=>	*I measure my body temperature at least twice every week*
wash_hands_many_times	=>	*I wash my hands with soap and water regularly*
hand_sanitizer	=>	*I use a hand sanitizer regularly*
touching	=>	*I avoid touching my face (eyes, nose and mouth)*
other	=>	*Other (please specify)*

Q: Please specify other protective measures:

Type: text

A: text input

Visible if

Q:	A:
:input[name="33_which_protective_measure_are_you_using_multiple_options_possi[other]"]	- checked => 1

Q: Have you been tested for COVID-19? (*)

Type: choice

A: one of the following:

yes	=>	*Yes*
no	=>	*No*

Q: Why were you tested for COVID-19? (*)

Type: choice

A: one of the following:

symptoms	=>	*Because I had symptoms of the disease*
contact	=>	*Because I was in contact with people who had the disease*
mass_testing	=>	*Because there was mass COVID-19 testing in my community*
work	=>	*Because COVID-19 testing was recommended or obligatory in my place of work*
paid	=>	*Because I personally wanted to know my COVID-19 status, so I paid for a test in a private laboratory*
other	=>	*For other reasons*

Visible if

Q:	A:
What was the result of the test?	- value => positive

Q: What was the result of the test? (*)

Type: choice

A: one of the following:

positive	=>	*Positive*
negative	=>	*Negative*
do_not_know	=>	*Do not know*

Visible if

Q:	A:
Have you been tested for COVID-19?	- value => yes

Q: If you tested positive for COVID-19, were you hospitalised? (*)

Type: choice

A: one of the following:

yes	=>	*Yes*
no	=>	*No*

Visible if

Q:	A:
What was the result of the test?	- value => positive

Q: According to you, what is the risk that you got infected with the coronavirus at some point during this epidemic?(*)

Type: choice_scale

A: On a scale of 1 to 5

1	=>	*1=Very little or no risk*
2	=>	*2=Little risk*
3	=>	*3=Moderate risk*
4	=>	*4=High risk*
5	=>	*5=Very high risk*

Visible if

Q:	A:
Have you been tested for COVID-19?	- value => no

Q: In case a COVID-19 vaccine is developed and approved, would you be willing to receive it? (*)

Type: choice

A: one of the following:

yes	=>	*Yes*
no	=>	*No*
dont_know	=>	*I don't know*

HIV follow-up after lockdown

Q: Please specify the anti-HIV treatment you take (name of medication, brand) (*)

Type: text

A: text input

Q: Have you visited a medical doctor or a health facility for your routine HIV follow-up during the last month?(*)

Type: choice

A: one of the following:

yes	=>	*Yes*
no	=>	*No*

Q: Were you able to refill you anti-HIV medications last month? (*)

Type: choice

A: one of the following:

yes	=>	*Yes, I got my refill without any problem*
no_stockout	=>	*No, because my drugs were not available at the pharmacy/clinic*
no_prescription	=>	*No, because I was unable to obtain my doctor's prescription*
no_restriction	=>	*No, because I could not go out due to COVID-19 restrictions*
na	=>	*Not applicable*

Q: Were you able to consult with your HIV physician face-to-face last month? (*)

Type: choice

A: one of the following:

yes	=>	*Yes, I met with my physician*
no_busy	=>	*No, because they are very busy with non-HIV patients, including COVID-19*
no_phone	=>	*No, I consulted by phone / online*
no_restrictions	=>	*No, because I could not go out due to COVID-19 restrictions*
na	=>	*Not applicable*

Q: How satisfied are you with the HIV care provided by your follow-up clinic and doctors during the COVID-19 epidemic period? (*)

Type: choice_scale

A: On a scale of 1 (very unsatisfied) to 5 (very satisfied)

1	=>	*Very unsatisfied*
2	=>	*Unsatisfied*
3	=>	*Neither satisfied nor unsatisfied*
4	=>	*Satisfied*
5	=>	*Very satisfied*

Q: (OPTIONAL) Can you briefly explain the reason for your level of satisfaction?

Type: text

A: text input

Q: Compared to the period before COVID-19 restrictions, how do you relate with healthcare workers now?(*)

Type: choice_scale

A: I trust healthcare workersI trust the healthcare system

more	=>	*More than before*
less	=>	*Less than before*
same	=>	*Same as before*

Q: Do you feel your HIV services have gotten back to normal (as before the COVID-19 epidemic)?(*)

Type: choice

A: one of the following:

yes	=>	*Yes*
no	=>	*No*

Q: How worried are you about getting exposed/infected with COVID-19 when going back to your clinic for follow-up visits/refills? (*)

Type: choice_scale

A: On a scale of 1 (not worried) to 5 (extremely worried)

1	=>	*Not at all worried*
2	=>	*A little worried*
3	=>	*Moderately worried*
4	=>	*Very worried*
5	=>	*Extremely worried*

Q: Do you think you may be needing any additional support as a consequence of the COVID-19 crisis on your psychological and social well-being? (*)

Type: choice

A: one of the following:

yes	=>	*Yes*
no	=>	*No*

Q: Is any form of psychosocial support available at your clinic? (*)

Type: choice

A: one of the following:

yes	=>	*Yes*
no	=>	*No*

Q: Do you have any suggestions for HIV care during this period when the COVID-19 restrictions are being relaxed?

Type: text

A: text input

Q: I fully understand what this study is about, and I consent to participate. All the information I provide can be used by researchers to better understand coronavirus disease and its effect on persons living with HIV infection. (*)

Type: checkbox

A: checkbox

Q: (OPTIONAL) I consent to be re-contacted by the researchers for feedback and follow-up questions on this topic, and I register my email address for this purpose. My email address will only be used to anonymously re-contact me via an automatic email. It will not be visible or accessible to the research team.

Type: checkbox

A: checkbox

Q: Please provide your email address:

Type: text

A: text input

Visible if

Q:	A:
(OPTIONAL) I consent to be re-contacted by the researchers for feedback and follow-up questions on this topic, and I register my email address for this purpose. My email address will only be used to anonymously re-contact me via an automatic email. It will not be visible or accessible to the research team.	- checked => 1
