# Supplementary material for: Follow-Up Survey of the Impact of COVID-19 on People Living with HIV during the Second Semester of the Pandemic
Source: Int J Environ Res Public Health. 2021 Apr 27;18(9):4635. doi: 10.3390/ijerph18094635 (PMC8123847; doi:10.3390/ijerph18094635)
Supplement: Supplementary file 1 [file ijerph-18-04635-s001.zip › Supplementary S2.pdf]

SUPPLEMENTARY S2

Country of residence of participants, country classification, and number of respondent per country

Country	Geographical location[a]	Classification[b]	Number of respondents	Percentage of study population
Algeria	Africa	LMIC	1	0.4%
Argentina	Latin America	LMIC	1	0.4%
Belarus	Eastern Europe	LMIC	2	0.8%
Belgium	Western Europe	HIC	82	33.2%
Bermuda	North America	LMIC	1	0.4%
Brazil	Latin America	LMIC	83	33.6%
Colombia	Latin America	LMIC	1	0.4%
France	Western Europe	HIC	14	5.7%
Georgia	Eastern Europe	LMIC	2	0.8%
Germany	Western Europe	HIC	1	0.4%
Greece	Western Europe	HIC	1	0.4%
Ireland	Western Europe	HIC	1	0.4%
Italy	Western Europe	HIC	2	0.8%
Kazakhstan	Asia	LMIC	2	0.8%
Luxembourg	Western Europe	HIC	2	0.8%
Moldovia	Eastern Europe	LMIC	2	0.8%
Netherlands	Western Europe	HIC	3	1.2%
Portugal	Western Europe	HIC	1	0.4%
Romania	Eastern Europe	LMIC	3	1.2%
Russia	Eastern Europe	LMIC	28	11.3%
South Africa	Africa	LMIC	1	0.4%
Spain	Western Europe	HIC	4	1.6%
Switzerland	Western Europe	HIC	1	0.4%
Turkey	Western Europe	LMIC	1	0.4%
UK	Western Europe	HIC	3	1.2%
Ukraine	Eastern Europe	LMIC	4	1.6%

a. United Nations DGACM. *https://www.un.org/depts/DGACM/RegionalGroups.shtml*

b. World Bank Country and Lending Groups – World Bank Data Help Desk. *https://datahelpdesk.worldbank.org/knowledgebase/articles/906519-world-bank-country-and-lending-groups*
