# Supplementary material for: Follow-Up Survey of the Impact of COVID-19 on People Living with HIV during the Second Semester of the Pandemic
Source: Int J Environ Res Public Health. 2021 Apr 27;18(9):4635. doi: 10.3390/ijerph18094635 (PMC8123847; doi:10.3390/ijerph18094635)
Supplement: Supplementary file 1 [file ijerph-18-04635-s001.zip › Supplementary S3.pdf]

Supplementary S3

Comparison of respondents from the four most represented countries, *n* = 207

Characteristics	Belgium *n* = 82	Brazil *n* = 83	France *n* = 14	Russia *n* = 28	*p*-value
Age, years: mean (SD)	50.8 (11.5)	37.9 (11.8)	53.9 (12.3)	37.7 (8.3)	< 0.001
**Sex: *n* (%)**					< 0.001
Male	67 (81.7%)	61 (73.5%)	13 (92.9%)	12 (42.9%)	
Female	14 (17.1%)	20 (24.1%)	1 (7.1%)	16 (57.1%)	
Other	1 (1.2%)	2 (2.4%)	0 (0%)	0 (0%)	
**Country classification based on World Bank**	HIC	LMIC	HIC	LMIC	NA
**Highest education level: *n* (%)**					NA
Primary	2 (2.4%)	2 (2.4%)	0 (0%)	12 (42.9%)	
Secondary	39 (47.6%)	16 (19.3%)	4 (28.6%)	8 (28.6%)	
Undergraduate	25 (30.5%)	27 (32.5%)	7 (50.0%)	2 (7.1%)	
Post-graduate	16 (19.5%)	38 (45.8%)	3 (21.4%)	6 (21.4%)	
**Religion: *n* (%)**					NA
Christian	40 (48.8%)	44 (53.0%)	2 (14.3%)	16 (57.1%)	
Muslim	1 (1.2%)	1 (1.2%)	0 (0%)	3 (10.7%)	
Other	2 (2.4%)	22 (26.5%)	1 (7.1%)	4 (14.3%)	
None	39 (47.6%)	16 (19.3%)	11 (78.6%)	5 (17.9%)	
**Marital status: *n* (%)**					NA
Single	34 (41.5%)	50 (60.2%)	6 (42.9%)	5 (17.9%)	
Stable relationship	13 (15.9%)	11 (13.3%)	3 (21.4%)	1 (3.6%)	
Cohabitation	9 (11.0%)	10 (12.0%)	2 (14.3%)	6 (21.4%)	
Married	10 (12.2%	8 (9.6%)	3 (21.4%)	10 (35.7%)	
Divorced	13 (15.9%)	4 (4.8%)	0 (0%)	2 (7.1%)	
Other	3 (3.7%)	0 (0%)	0 (0%)	4 (7.3)	
Visited health facility or HIV clinic during the last month for routine HIV follow-up: *n* (%)	49 (59.8%)	39 (47.0%)	8 (57.1%)	17 (60.7%)	0.351
Received flu vaccination during the past 12 months: *n* (%)	70 (85.4%)	72 (86.7%)	11 (78.6%)	4 (14.3%)	< 0.001
**COVID-19 test results: *n* (%)**					0.220
Positive	2/19 (10.5%)	5/14 (35.7%)	2/9 (22.2%)	0/8 (0%)	
Negative	17/19 (89.5%)	9/14 (64.3%)	7/9 (77.8%)	8/8 (100%)	
**Willing to take COVID-19 vaccine: *n* (%)**					NA
Yes	55 (67.1%)	81 (97.6%)	12 (85.7%)	9 (32.1%)	
No	5 (6.1%)	0 (0%)	0 (0%)	13 (46.4%)	
Don't know	22 (26.8%)	2 (2.4%)	2 (14.3%)	6 (21.4%)	
Depression, PHQ-2 score≥3: *n* (%)	16 (19.5%)	34 (41.0%)	1 (7.1%)	7 (25.0%)	0.005
Anxiety, GAD-2 score≥3: *n* (%)	14 (17.1%)	33 (39.8%)	2 (14.3%)	9 (32.1%)	0.007

HIC: High income country
LMIC:Low- and Middle-income country
NA: Not applicable
